# The Paradigm Shift to Non-Treatment of Asymptomatic Bacteriuria

**DOI:** 10.3390/pathogens5020038

**Published:** 2016-04-19

**Authors:** Lindsay E. Nicolle

**Affiliations:** Department of Internal Medicine and Medical Microbiology, University of Manitoba, Health Sciences Centre, Room GG443—820 Sherbrook Street, Winnipeg, MB R3A 1R9, Canada; lnicolle@hsc.mb.ca; Tel.: +1-204-787-7029; Fax: +1-204-787-4826

**Keywords:** bacteriuria, asymptomatic bacteriuria, asymptomatic urinary tract infection, bacterial interference

## Abstract

Asymptomatic bacteriuria, also called asymptomatic urinary infection, is a common finding in healthy women, and in women and men with abnormalities of the genitourinary tract. The characterization and introduction of the quantitative urine culture in the 1950s first allowed the reliable recognition of asymptomatic bacteriuria. The observations that a substantial proportion of patients with chronic pyelonephritis at autopsy had no history of symptomatic urinary infection, and the high frequency of pyelonephritis observed in pregnant women with untreated asymptomatic bacteriuria, supported a conclusion that asymptomatic bacteriuria was harmful. Subsequent screening and long term follow-up programs for asymptomatic bacteriuria in schoolgirls and women reported an increased frequency of symptomatic urinary tract infection for subjects with asymptomatic bacteriuria, but no increased morbidity from renal failure or hypertension, or increased mortality. Treatment of asymptomatic bacteriuria did not decrease the frequency of symptomatic infection. Prospective, randomized, comparative trials enrolling premenopausal women, children, elderly populations, patients with long term catheters, and diabetic patients consistently report no benefits with antimicrobial treatment of asymptomatic bacteriuria, and some evidence of harm. Several studies have also reported that antimicrobial treatment of asymptomatic bacteriuria increases the short term risk of pyelonephritis. Current investigations are exploring the potential therapeutic intervention of establishing asymptomatic bacteriuria with an avirulent *Escherichia coli* strain to prevent symptomatic urinary tract infection for selected patients.

## 1. Asymptomatic Bacteriuria

Asymptomatic bacteriuria is the isolation of bacteria in quantitative counts consistent with growth within the bladder or kidneys from an appropriately collected urine specimen in an individual with no acute signs or symptoms referable to the genitourinary tract [1]. The appropriate quantitative count is ≥10^5^ cfu/mL for voided specimens. For women, two consecutive specimens obtained at least several days apart are recommended to confirm asymptomatic bacteriuria. However, for many women with an initial positive culture which is not subsequently confirmed, transient bacteriuria rather than contamination seems likely. For urine specimens obtained using an in and out catheter, including intermittent catheterization, bacteriuria is identified by ≥10^2^ cfu/mL. For patients with a chronic indwelling catheter ≥10^5^ cfu/mL is the appropriate criteria, as catheter biofilm contaminates urine specimens collected through the indwelling catheter [2].

## 2. Prevalence

Asymptomatic bacteriuria is common in many populations, but the prevalence is highly variable (Table 1) [3]. The prevalence increases with age; by 80 years 20% of healthy women resident in the community have a positive urine culture. Asymptomatic bacteriuria is rare in healthy men prior to 60 years of age, but for elderly men in the community prevalence rates of 3.6%–19% are reported [3]. Some populations with functional or structural genitourinary abnormalities, including patients with indwelling urinary devices, have a very high prevalence of bacteriuria [3]. Patients with spinal cord injury managed with intermittent catheterization or, in men, with sphincterotomy, have a prevalence of bacteriuria of 50% [3,4]. Individuals with chronic indwelling catheters are virtually always bacteriuric [2]. The prevalence of bacteriuria is increased in women with diabetes compared with non-diabetic women, primarily attributable to neurological complications of diabetes which impair bladder voiding, but is not increased for diabetic men [3,4].

## 3. Early Observations

Asymptomatic bacteriuria was first identified in the mid-1950s, when Kass and others described the use of the quantitative urine culture to differentiate true bacteriuria from contamination of voided urine specimens. In an initial study [5], paired specimens collected by in and out catheter from 67 asymptomatic, untreated, women identified one group where both specimens had bacteriuria with quantitative counts >10^3^ cfu/mL and another with lower counts. From 30% to 40% of the women with repeated high quantitative counts also had pyuria (>5 white blood cells/high power field). Pyuria was not present in specimens from women with lower quantitative counts.

Autopsy series reported in 1933, 1939, and 1957 identified chronic pyelonephritis as a common cause of renal failure [6]. However, only 0%–30% of individuals with chronic pyelonephritis at autopsy had a diagnosis of urinary tract infection while alive. As asymptomatic bacteriuria was now recognized to be common in some populations and frequently associated with inflammation in the urinary tract, a hypothesis that asymptomatic bacteriuria contributed substantially to the burden of chronic renal failure and death attributable to chronic pyelonephritis seemed reasonable. In addition, studies which enrolled pregnant women in the 1960s consistently reported an association of persistent asymptomatic bacteriuria with pyelonephritis later in pregnancy [3]. Bacteriuria was also associated with premature labor, intrauterine growth retardation, and increased neonatal death [3]. Treatment of asymptomatic bacteriuria in early pregnancy prevented subsequent pyelonephritis.

Thus, Kass wrote, in 1962, “there is now clear evidence that bacteriuria is one of the commonest human infections, that it may be chronic and persistent, that it may influence structure and function outside of the urinary tract, and that it plays an important role in disease from the cradle to the grave—from prematurity to hypertension and renal failure” [7].

## 4. Long Term Prospective Studies in Non-Pregnant Populations

A population-based screening study in Sweden identified and followed adult women with asymptomatic bacteriuria, initially with treatment of the bacteriuria. By 24 months follow-up after antimicrobial treatment, bacteriuria recurred in 52% [8]. At 15 [9] and 28 [10] years follow-up, women with bacteriuria at enrollment had an increased frequency of recurrent bacteriuria irrespective of antimicrobial treatment, but no excess hypertension, renal failure, or increased mortality. In the US, Savage followed 63 five-year old girls with asymptomatic bacteriuria for up to 4 years [11]. Lindberg, in Sweden, followed 116 children aged 7–15 years of age for up to 3 years [12]. In these cohorts, antimicrobial treatment did not decrease the prevalence of bacteriuria, frequency of pyelonephritis, or progression to renal scars. The authors concluded that untreated asymptomatic bacteriuria was not harmful for schoolgirls. It is also now recognized that the histologic finding of “chronic pyelonephritis” is a non-specific end stage attributable to many inflammatory disorders of the kidney and not a consequence of asymptomatic bacteriuria. Urinary infection, by itself, is a rare cause of renal failure.

Asscher *et al.* [13] reported an early placebo controlled trial of treatment of asymptomatic bacteriuria in adult women aged 20–65 years. Women were randomized to receive either nitrofurantoin or placebo; subjects not cured with nitrofurantoin were retreated with ampicillin. At 4 days after nitrofurantoin treatment, 20% of women remained bacteriuric. After one year, 45% of nitrofurantoin and 63% of placebo recipients had bacteriuria, and 37% and 36%, respectively, experienced symptomatic urinary tract infection. The authors concluded that treatment of asymptomatic bacteriuria had no benefits for these women.

Subsequently, randomized clinical trials of treatment of asymptomatic bacteriuria in children, non-pregnant women, elderly persons, spinal cord injury patients, persons with diabetes, and individuals with chronic catheters all reported that treatment of asymptomatic bacteriuria did not decrease the frequency of symptomatic urinary tract infection [1,14]. In addition, no other short or long term benefits of treatment were identified. Reinfection following therapy was frequent, and often associated with isolation of an organism of increased resistance. However, pregnant women remain a unique population. Treatment of asymptomatic bacteriuria identified in early pregnancy has been consistently documented to decrease the frequency of pyelonephritis later in pregnancy, with improved fetal outcomes [3].

## 5. Clinical Guidelines

Evidence based guidelines (Table 2) recommend screening and treatment of asymptomatic bacteriuria only for pregnant women or as surgical prophylaxis prior to selected urological procedures where mucosal trauma is expected [1]. There is strong evidence to support recommendations to not treat asymptomatic bacteriuria and, thus, not to screen for bacteriuria, in premenopausal non-pregnant women, diabetic women, elderly institutionalized men and women, and patients with long or short term indwelling urethral catheters. Evidence is of lower quality for older persons in the community or patients with spinal cord injury, but also supports a recommendation not to treat asymptomatic bacteriuria. These guidelines could not address patients with neutropenia, renal transplant patients, or patients undergoing elective orthopedic surgical procedures because of insufficient evidence.

## 6. Recent Studies

Observations from more recent studies continue to support the guideline recommendations. A prospective, observational study of women with type 1 and type 2 diabetes, 17% of whom had asymptomatic bacteriuria at enrollment, reported bacteriuria was not associated with a relative or absolute decrease in creatinine clearance during 6 years follow-up [14]. A recent Cochrane collaboration reviewed outcomes following antimicrobial treatment of covert bacteriuria in children [15]. Identification of bacteriuria was associated with bacteriuria at 6 months or at 4.5 years follow-up. However, treatment of asymptomatic bacteriuria did not decrease the incidence of symptomatic infection or influence renal growth.

Green *et al.* [16] described a retrospective cohort study of patients within one year following renal transplant who were treated or not treated for asymptomatic bacteriuria based on physician decision. The primary outcome of hospitalization for symptomatic urinary tract infection or greater than 25% reduction in the estimated glomerular filtration rate occurred in 18.2% of treated and 5.6% of not treated subjects. This study is subject to bias as, presumably, seriously ill patients would be more likely to be treated. Other observational studies of renal transplant patients have also reported that asymptomatic bacteriuria does not predict decline in renal function or increased risk for severe infection, graft loss, or mortality [4].

Sousa *et al.* [17] recently reported a retrospective case control study of treatment of asymptomatic bacteriuria, based on physician preference, prior to elective prosthetic joint insertion. Consistent with previous reports, asymptomatic bacteriuria identified pre-operatively was significantly associated with post-operative prosthetic joint infection. However, prosthetic joint infection was similar for patients with or without preoperative treatment of asymptomatic bacteriuria (treated 3.9%, untreated 4.7%). The urine was not the source of the infecting organisms, as gram positive organisms were usually isolated from the infected prosthetic joint and gram negative organisms from the urine. Thus, evidence supports a recommendation not to screen for or treat asymptomatic bacteriuria in orthopedic patients prior to elective prosthetic joint insertion.

## 7. Is Asymptomatic Bacteriuria Beneficial?

The early randomized trial in adult women reported by Asscher *et al.* [13] observed that 12 of 15 subjects with reinfection were symptomatic, and only 20 of 44 with persisting or relapsing bacteriuria (*p* = 0.05). As most reinfections occurred in the nitrofurantoin group, this suggested that treatment of bacteriuria was associated with an increased risk of symptomatic reinfection.

A Swedish report by Hansson *et al.*, describes outcomes following concomitant antimicrobial therapy given for group A streptococcal pharyngitis in schoolgirls enrolled in a long term prospective study of asymptomatic bacteriuria [18]. Episodes of symptomatic urinary infection were observed following penicillin therapy, an antimicrobial with sufficient renal excretion to inhibit *E. coli* in the urine. For 46 pharyngitis episodes, five courses of penicillin therapy were followed by eradication of bacteriuria, 34 by persistent or recurrent bacteriuria, and seven (15%) by symptomatic urinary infection within 30 days, with six presenting as pyelonephritis and one cystitis. For all symptomatic episodes, a strain of *E. coli* distinct from the pre-penicillin strain was isolated. When treatment for pharyngitis was changed to erythromycin, an antimicrobial which does not impact bacteriuria, there was uniform persistent or recurrent bacteriuria with the pre-therapy *E. coli* strain following therapy, and no symptomatic episodes. The authors concluded that eradication of bacteriuria by concomitant antimicrobial therapy was a risk for development of symptomatic infection in these girls.

A prospective, randomized placebo controlled trial of treatment of asymptomatic bacteriuria in diabetic women reported a similar frequency of pyelonephritis in treated or untreated subjects during three years follow-up [19]. However, five of six episodes of pyelonephritis in the treatment group occurred within four months of initial treatment of bacteriuria, and only two of 11 in the placebo group. Observations in this study also suggest that eradication of stable strains causing long term asymptomatic bacteriuria increases the short term risk of pyelonephritis.

Cai *et al.* [20] recently reported a non-blinded trial of outcomes following antimicrobial therapy given to young women presenting to a sexually transmitted infection clinic with bacteriuria identified and at least one episode of symptomatic urinary infection in the past year. Women treated for bacteriuria had a substantially higher frequency of recurrent symptomatic urinary infection in the year following treatment. However, a high proportion of bacteria isolated were *Enterococcus faecalis*, which is usually a contaminant, so the generalizability of these observations is uncertain.

Several investigators have explored the use of “bacterial interference” to prevent symptomatic episodes in patients with underlying genitourinary abnormalities and frequent symptomatic urinary tract infection [21]. This intervention is based on the hypothesis that bacteriuria with a non-pathogenic *E. coli* strain prevents infection with more virulent strains which are more likely to cause symptomatic infection. Preliminary studies suggest this approach may benefit selected patients in whom colonization can be established and maintained.

## 8. Conclusions

During the past 50 years, the paradigm for asymptomatic bacteriuria has shifted from being a harmful clinical finding requiring identification and treatment, to a benign observation requiring no management in most non-pregnant subjects and now, potentially, to being beneficial for some patients. The optimal management of bacteriuria for most populations is well characterized, although operational issues remain in implementing a “do not treat” approach for many patients. The potential role of asymptomatic bacteriuria as a strategy to prevent symptomatic infection in selected individuals is being investigated.

## Figures and Tables

**Table 1:** Prevalence of asymptomatic bacteriuria reported in different populations.

		Proportion with Bacteriuria
Healthy, pre-menopausal women		1.0%–5.0%
Pregnant women		1.9%–9.5%
Postmenopausal women (50–70 years)		2.8%–8.6%
Diabetic:	Women	9%–27%
	Men	0.7%–11%
Elderly in community:	Women	10.8%–16%
	Men	3.6%–19%
Elderly in long term care:	Women	25%–50%
	Men	15%–40%
Spinal cord injury		50%
Chronic indwelling catheter		100%

**Table 2:** IDSA Recommendations for asymptomatic bacteriuria in adults [1].

**Screening and Treatment Recommended (Level of Evidence)**
pregnant women (AI)
transurethral resection of the prostate (AI)
other traumatic urologic procedures (AIII)
**Screening and Treatment not Recommended (Level of Evidence)**
premenopausal, non-pregnant women (AI)
diabetic women (AI)
older persons living in the community (AII)
elderly institutionalized residents (AI)
persons with spinal cord injury (AII)
catheterized patients (AI)
